# TFCdetector: Multivariable time series anomaly detection with time-frequency constraints

**DOI:** 10.1371/journal.pone.0361145

**Published:** 2026-10-28

**Authors:** Jinyu Fan, Jianfei Zhao, Jun Ma, Yuxi Cai

**Affiliations:** 1 College of Physics and Electronic Information Engineering, Qinghai Normal University, Xining, China; 2 College of Computer Technology and Application, Qinghai University, Xining, China; 3 College of Computer Science and Technology, Xinjiang University, Urumqi, China; Universitas Mercatorum, ITALY

## Abstract

The multivariate time series collected from numerous sensors exhibit complex interwoven relationships, arising from both the coupling among different components and the temporal dependencies of fluctuations within each component. Disentangling these relationships remains a persistent challenge for the time series community. Existing reconstruction-based methods attempt to unravel the intertwined relationships through sequence decomposition and multi-scale modeling. However, the decomposed components remain mixtures of complex latent factors. Moreover, most methods overlook anomalies present in the training set, which inadvertently causes the model to develop a certain affinity toward anomalous patterns. To address these limitations, we propose TFCdetector. Motivated by the intrinsic properties of anomalies, TFCdetector leverages time-frequency domain analysis to explicitly suppress the model’s representation learning of anomalies across diverse granularities and components. To further constrain the model’s learning of anomalies, we propose an energy-correlation constraint loss that operates on both segment-level energy and sequence-level correlation. Notably, the adaptively updated memory storage block enhances the expression of normal patterns by leveraging the affinity between normal patterns in the input sequence and the memory storage block. Furthermore, the meticulously designed twin frequency attention enables the model to dynamically select local or global frequency information beneficial for the task. Extensive experiments on 1 synthetic dataset and 7 real-world datasets show that TFCdetector achieves competitive results in multivariate time series detection tasks.The implementation code is available at https://github.com/FanJY3/TFCdetector.

## 1. Introduction

Multivariate time series data gathered from numerous sensors provides a numerical overview of a system’s status, and it is widely used in fields like transportation, weather, and aerospace [1–4]. Efficient detection of extraordinary patterns in systems is crucial for ensuring smooth operation. Multivariate time series anomaly detection, based on the analysis of historical data, detects unusual pattern in the sequence, which is essential for proactive system maintenance and risk management.

With the rapid advancement of sensor and communication technologies, the volume of multivariate time series (MTS) data has grown exponentially, rendering traditional anomaly detection techniques [5–7] inadequate for efficiently processing such massive and complex sequential data. MTS data collected from multiple sensors exhibit intricate interdependent relationships, stemming from both the spatial correlations across different variables and the temporal dynamics within each individual variable. Modeling these complex dependencies continues to be a formidable challenge for the time series community [8]. Deep learning-based methods have emerged as the most promising solution, owing to the powerful representation capabilities of neural networks. However, given that anomalies are typically rare and subtle, manual labeling is prohibitively time-consuming, labor-intensive, and prone to mislabeling or omission. This makes it exceptionally difficult to train supervised deep anomaly detection models. Consequently, unsupervised learning [9,10] has gained prominence, as it derives supervisory signals directly from the data itself. These deep learning approaches can be broadly categorized into prediction-based [11,12] and reconstruction-based methods [13,14]. Prediction-based methods leverage knowledge learned from historical data to forecast future time steps, identifying anomalies based on prediction errors and predefined thresholds. However, these methods inherently assume that all samples in the training set are normal, overlooking the potential interference of anomalies or noise. This vulnerability can lead to degraded performance, as the model may inadvertently learn anomalous patterns. Reconstruction-based methods, typically exemplified by autoencoders, operate on the assumption that anomalous samples are more difficult to reconstruct in a low-dimensional latent space than normal ones. Due to the influence of anomalies or noise, coupled with the powerful encoding and decoding capabilities of autoencoder [15], especially the encoder obtaining the relevant patterns of anomalies, abnormal inputs may also have lower reconstruction errors. Additionally, the L-norm reconstruction loss only measures the overall quality of reconstruction, reducing the contribution of locally anomalous parts that are more difficult to reconstruct. To disentangle the intermingled components within time series, decomposition and sampling techniques have been widely explored. Decomposition-based methods [16,17] typically separate sequences into periodic, trend, and residual components, while sampling-based approaches, such as down-sampling [18,19] or multi-scale techniques [20,21], generate various sub-modes through sampling operations. However, the resulting components from either decomposition or sampling remain inherently mixed. For instance, the periodic term is often a superposition of multiple frequency cycles, and down-sampled sub-segments still contain entangled mixtures of complex components. This inherent mixing makes it challenging for models to extract clean and discriminative normal patterns.

Time-frequency domain analysis [22,23] offers a promising alternative by decomposing sequences into non-overlapping frequency components, thereby unraveling these entangled relationships. The most closely related work, MEGA [24], employs the discrete wavelet transform (DWT) to decompose sequences into multiple frequency sub-bands before reconstructing them. Despite its innovation, MEGA suffers from three critical limitations. First, it extracts patterns from individual sub-bands in isolation, neglecting the cross-granularity relationships both within and across sub-bands. Second, MEGA relies on a sequence-level reconstruction loss within an autoencoder-style framework to constrain anomaly reconstruction, yet it overlooks the autoencoder’s inherent over-generalization problem-its excessive representational capacity allows anomalous inputs to be reconstructed with deceptively low errors. Third, this relatively coarse sequence-level reconstruction loss dilutes the contribution of localized anomalous segments, as the overwhelming presence of normal patterns dominates the overall reconstruction error, further degrading detection performance.

To overcome these limitations, we propose TFCdetector, a novel time-frequency constrained framework that explicitly addresses anomalies from their intrinsic characteristics. Our approach leverages the wavelet transform-grounded in rigorous mathematical theory to decompose the original time series into non-overlapping sub-band spaces. To prevent the model from learning anomalous patterns at multiple granularities within each sub-band, we design an adaptive multi-granularity filtering block and introduce a novel energy-correlation constraint loss, which jointly suppress anomaly representation from both segment-level and sequence-level perspectives. Furthermore, a memory storage block enhances the representation of normal patterns by capturing the affinity between normal patterns in the input sequence and those stored in the memory, thereby effectively mitigating anomaly contamination. Finally, our meticulously designed twin frequency attention mechanism enables the model to dynamically select task-relevant frequency information at both local and global scales, significantly enhancing its ability to capture discriminative features.

The key contributions of this work are summarized as follows:

(1) Starting from the mutual relationships among anomalies, we propose the TFCdetector, which combines adaptive multi-granularity filtering and energy-correlation constraint loss to suppress the model’s learning for anomalies at different granularities in each sub-band space from both the segment level and the sequence level.
(2) The twin frequency attention mechanism is proposed, which constructs the network’s ability to acquire local and global information from the perspective of the frequency domain. Meanwhile, the adaptively updated memory storage block utilizes the affinity between normal patterns to strengthen the representation of normal patterns in the input sequence.
(3) Extensive experiments on 7 real-world datasets and 1 synthetic dataset demonstrate that TFCdetector outperforms other advanced anomaly detection algorithms overall. Fine-grained analysis, effectiveness evaluation, and parameter sensitivity analysis demonstrate the effectiveness of key components in TFCdetector, enabling it to perform robust performance across all datasets.

## 2. Related work

### 2.1 Anomaly detection based on prediction

Prediction-based methods forecast future one or more consecutive discrete values based on historical patterns derived from past data, and determine the anomaly degree of time points based on prediction errors. Literatures [25,26] use RNN as the prediction model to predict future time steps, and the mean squared error between the predicted values and the actual values is used as the criterion for judging anomalies. Lai [27] attempts to combine CNN and RNN to capture the local and global dependency patterns of the sequence for better representing multivariate time series. Kravchik [28] applies one-dimensional convolutional networks to industrial control systems and achieves advanced anomaly detection accuracy. Zhang [29] uses a memory network composed of linear and nonlinear predictions to capture temporal dependencies and uses maximum mean discrepancy to measure the difference between the latent variables of the autoencoder and the Gaussian distribution to describe the spatial dependencies of the sequence. However, whether this Gaussian prior is applicable to other data is open to question. Xiao [30] jointly trains two complementary proxy tasks and uses a compression storage module to maintain normal patterns, avoiding unexpected generalization to anomalous inputs. Pan [31] uses a block masking mechanism to enhance the predictor’s robustness to anomalous data and a maximum masking self-attention to reduce noise dependencies between irrelevant sensors, while it ignores the effect of entangled temporal relations for learning the normal pattern.

### 2.2 Anomaly detection based on reconstruction

Reconstruction models typically assume that anomalous samples are difficult to reconstruct from the latent space, and they optimize the model by minimizing reconstruction error. Zong [32] optimizes the deep autoencoder and mixed model in an end-to-end manner to avoid the autoencoder falling into local optima. Malhotra [33] proposes an LSTM-based encoder-decoder network to learn the temporal representation of input time series. Variants of autoencoder, e.g., variational autoencoder [34] and denoising autoencoder, have also emerged. Su [35] proposes a recurrent convolutional encoding attention mechanism to extract discriminative features from chaotic time series, coupled with an enhanced empirical mode decomposition (EMD) method that reconstructs the original sequence through hierarchical high-to-low-frequency component separation, thereby suppressing high-frequency noise interference. Pan [36] incorporates diffusion process information into the self-attention mechanism to explicitly model the probabilistic distribution of diffusion dynamics across discrete temporal steps, thereby significantly improving anomaly detection effectiveness for diverse anomaly categories. Wang [37] conducts an in-depth study on variational autoencoder and points out that existing VAE-based models fail to capture heterogeneous periodic patterns and detailed trend patterns due to the lack of some frequency information. Based on this, FCVAE is proposed, bringing VAE-based methods back into focus. Liu [38] attempts to combine autoencoder with generative adversarial networks, robustly reconstructing data by jointly optimizing hidden feature matching and reconstruction in the generative adversarial network. Du [39] uses the autoencoder to generate pseudo-labels for the data to filter out possible anomalous samples, assisting the network in adversarial training. Song [15] embeds a memory mechanism in the autoencoder that can adaptively fuse global and local information in the sequence to improve the network’s representational capability. However, these methods can only measure the reconstruction as a whole, diminishing the impact of local anomalies that are more difficult to reconstruct.

### 2.3 Sequence analysis based on decomposition and sampling

Facing complex interdependent relationships entangled within multivariate time series, sequence decomposition or sampling has become an effective means to unravel these entanglements. Gao [16] first combines series decomposition with depth neural network to detect time series anomalies, decomposing time series into different periods, trends and other components, and then based on the decomposed components to detect anomalies. Zhong [40] analyzes time series data by learning to explicitly decompose and represent temporal patterns, and uses MLP to capture changes within and between blocks and channel correlations. Based on the periodic behavior of the sequence itself, Wu [8] embeds the intra-period and inter-period variations into the columns and rows of the 2D tensor, respectively, allowing sequence analysis to draw on more sophisticated processing techniques in the field of computer vision. Ge [41] uses two-dimensional singular value decomposition as representation method to capture the correlations between variables, and accurately extracts multiple modes of the sequence based on the subspace reconstruction strategy of sparse autoencoders and Gaussian mixture models. Based on variational inference, LaST [42] is proposed to unravel the seasonal and trend representations in the latent space from the perspectives of itself and input reconstruction. Wang [43] embeds trend decomposition and wavelet transform into the UNet network to explore local details and overall trends at different scales in the original sequence, however, the decomposed portion still contains a variety of components.

Methods based on sequence sampling capture multiple scale views of the original sequence through techniques like down-sampling and convolution kernels of different sizes, and acquire more powerful sequence representations. Liu [19] uses a recursive down-sampling interaction structure to iteratively extract and exchange information at multiple time resolutions, learning effective representations with enhanced predictability. Wang [20] and Luo [44] obtain multi-scale sequence information under different receptive fields through different convolution kernels to enhance the model’s expressive power. Yue [18] performs contrastive learning in a hierarchical manner to extract permutation-invariant representations of the sequence at different granularities. Cai [21] utilizes frequency domain analysis and adaptive graph convolution to capture the inter-sequence correlations of changes in time series at different time scales. Lyu [45] proposes a model-agnostic framework that can sequentially aggregate information from various scales without destroying sequence features. Deznabi [46] uses a deep learning model to model each sub-band signal after wavelet transform. Liu [47] combines the advantages of Fourier transform and wavelet transform from the frequency domain perspective to extract comprehensive time-frequency information from the signal. MEGA [24] ignores the interference of anomalies or noise although it utilizes the discrete wavelet transform to decompose the sequence into multi-frequency components and learns the normal pattern of the sequence by reconstruction.

## 3. Network framework

In this section, we first present the preliminaries in Section 3.1, including the problem formulation of time series anomaly detection, the attention mechanism, and the wavelet decomposition process. In Section 3.2, we outline the overall architecture and workflow of the proposed TFCdetector. Subsequently, Sections 3.3–3.5 elaborate on the design and implementation of each key component in detail. Finally, we introduce the training objectives and the anomaly scoring criterion.

### 3.1 Preparatory work

#### 3.1.1 Problem definition.

In the context of multivariate time series anomaly detection, given an input sequence $X_{\text{t}-l:t}\in R^{n\times l}$ representing observations within a sliding window, *n* denotes the number of variables, and l represents the length of the sequence. $X_{t}^{i}$ denotes the observation at the t-th time step on the i-th variable. The goal of time series anomaly detection is to determine $X_{t}^{i}$ whether is an anomaly based on the patterns inherent in the sequence.

#### 3.1.2 Self-attention.

The Transformer has made a significant impact in natural language processing and computer vision fields [48,49], largely due to its ability to effectively model long-range dependencies through self-attention. For an input sequence $X_{\text{t}-l:t}\in R^{n\times l}$, self-attention maps it into Queries $Q\in R^{K\times D}$, Keys $K\in R^{K\times D}$, and Values $V\in R^{K\times D}$ through linear projections. It dynamically captures the relationships between different positions in the sequence through the dot product between *Q* and *K*, and normalizes the attention scores into the range [0,1] using the *Softmax* function.


$$\begin{array}{c} Q=X\times W_{\text{q}},\;K=X\times W_{\text{k}},\;V=X\times W_{\text{v}} \end{array}$$ (1)

$$\begin{array}{c} \text{Attention}\left(Q,K,V\right)=\text{Softmax}\left(\frac{QK^{T}}{\sqrt{D}}\right)V \end{array}$$ (2)

Here, $W_{\text{q}}\in R^{D\times D}$,$W_{\text{k}}\in R^{D\times D}$, and $W_{\text{v}}\in R^{D\times D}$are learnable parameter matrices, *K* is the dimension of the embedded sequence, and *D* is the length of the embedded sequence. The self-attention mechanism can flexibly capture complex data relationships over long ranges within the sequence through this querying approach.

#### 3.1.3 Wavelet decomposition.

The Discrete Wavelet Transform (DWT) is a powerful technique for signal analysis. Unlike the Fourier Transform, which captures only frequency information, DWT simultaneously preserves both frequency and temporal characteristics, making it particularly effective for capturing local features in the time-frequency domain. Compared to decomposition-based or sampling-based methods, wavelet transform decomposes a sequence into non-overlapping representations across different frequency bands, thereby effectively avoiding the component mixing issue inherent in traditional decomposition approaches. Specifically, DWT employs a pair of low-pass and high-pass filters to decompose a sequence into low-frequency and high-frequency components. This process is then recursively applied to the low-frequency components based on the specified decomposition level, ultimately yielding sub-sequences that reside within distinct frequency bands.


$$\begin{array}{c} A_{j+\text{1}}(n)=\sum_{m}cA_{j}(\text{2}n+m)h(m),\;cD_{j+\text{1}}(n)=\sum_{m}cA_{j}(\text{2}n+m)g(m) \end{array}$$ (3)

Here, h(·) refers to the low-pass filter, and g(·) refers to the high-pass filter. $cA_{j+1}(\cdot )$ and $cD_{j+1}(\cdot )$ represent the low-frequency and the high-frequency components at level *j + 1*, respectively.

### 3.2 An overall review of the TFCdetector

Fig 1 illustrates the overall architecture of the proposed TFCdetector, which primarily comprises four key modules: an adaptive multi-granularity filtering block, a twin frequency attention mechanism, a standard Transformer encoder, and a memory storage block. Specifically, the input sequence is first decomposed via the Discrete Wavelet Transform into non-overlapping sub-sequences across different frequency bands. These sub-sequences are then fed into the adaptive multi-granularity filtering block. Coupled with the proposed energy-correlation constraint loss, this module effectively suppresses the model’s learning of anomalous patterns within distinct frequency bands. Subsequently, the twin frequency attention mechanism is employed to enhance the model’s ability to selectively integrate local and global frequency information. This is followed by a standard Transformer encoder and a memory storage block, which work in tandem to augment the representation of normal patterns. Finally, the sequence is reconstructed via a multi-layer perceptron.

**Fig 1:**
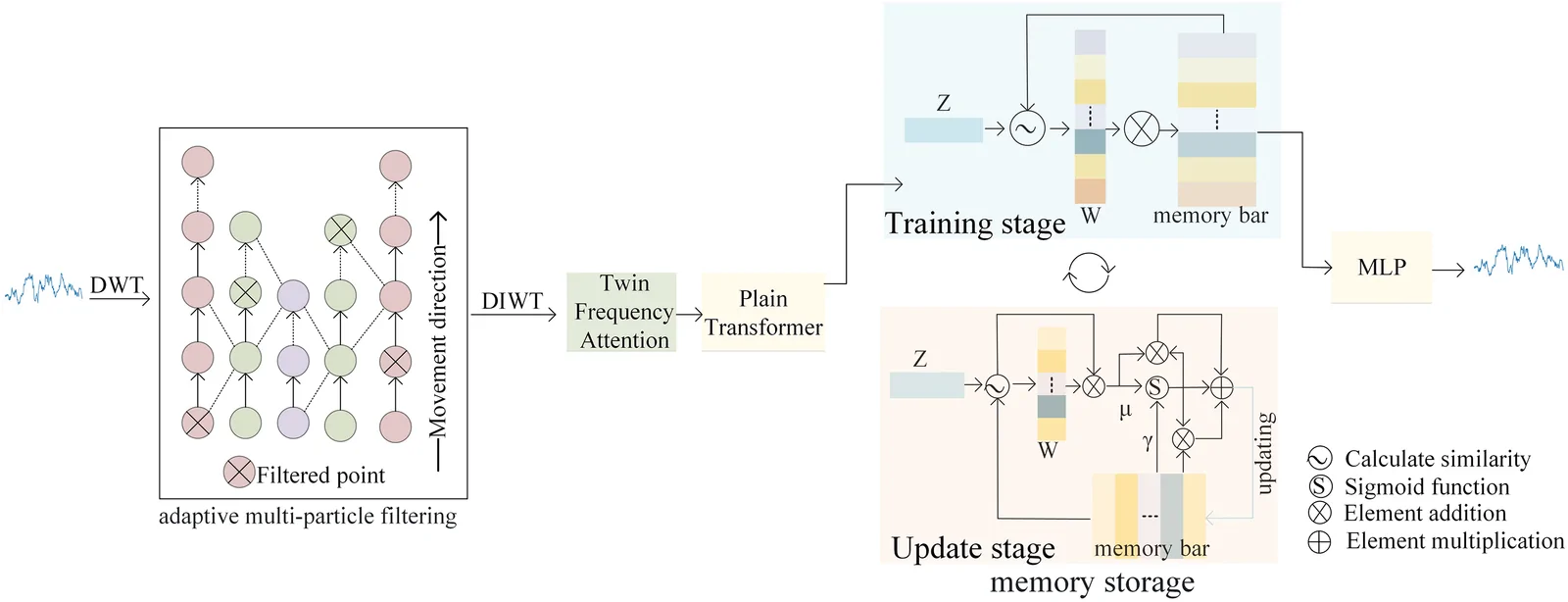
The overall diagram of the TFCdetector.

### 3.3 Adaptive multi-granularity filtering block collaborative energy-correlation constraint loss

#### 3.3.1 Adaptive multi-granularity filtering block.

To tackle the entangled dependencies within time series, we employ the Discrete Wavelet Transform to decompose the input sequences into sub-sequences spanning distinct frequency bands. Anomalies or noise that are masked by normal patterns in the time domain exhibit distinct manifestations in these decomposed sub-sequences. For instance, jitter or high-frequency noise is typically highlighted in high-frequency sub-sequences, whereas trend shifts tend to concentrate in the low-frequency region. Classical filtering algorithms, such as Gaussian or mean filters, not only require laborious hyper-parameter tuning but also lack the flexibility to adapt to the diverse anomalies present across different sequences. In contrast, convolutional kernels-conceptually similar to filters in signal processing-can be learned to extract task-relevant features while filtering out irrelevant information. Crucially, they can adaptively adjust filter weights based on the input data, enabling robust generalization across diverse datasets. Furthermore, incorporating multi-scale views of the sequence [20,21] has been shown to enhance the model’s sensitivity to anomalies. Motivated by these advantages, we leverage convolutional filters to construct the adaptive multi-granularity filtering block, which explicitly integrates multi-granularity information from the sub-sequences (see Fig 1).

Specifically, within the time-frequency domain, standard convolutional layers are applied to process sub-sequences across different frequency bands. Pooling operations are then utilized to obtain representations at varying sequence lengths, thereby generating multi-scale views for each frequency band. During network optimization, this adaptive block effectively filters out potential anomalies from sub-sequences of different frequencies under the guidance of the proposed energy-correlation constraint loss.

#### 3.3.2 Energy-correlation constraint loss.

Conventional reconstruction losses typically measure the overall reconstruction error, which inadvertently diminishes the contribution of localized anomalies that exhibit significant reconstruction errors to the total loss. Furthermore, models often struggle to effectively capture normal patterns that undergo abrupt changes, leading to the undesirable incorporation of these pattern deviations into the reconstruction error.

To address these limitations, we constrain the model’s representation of anomalies from two complementary perspectives: the energy distribution of sub-sequences across different frequency bands and the autocorrelation properties of the entire sequence. Anomalies that are inherently difficult to reconstruct tend to yield higher anomaly scores, manifesting as elevated amplitudes in the residual signal. Therefore, we constrain the model’s learning of local anomalies within sub-sequences from an energy perspective. Moreover, an effective filtering model should preserve essential features while ensuring that the residual signal exhibits minimal autocorrelation. Since anomalies typically display weak or negligible autocorrelation-unlike normal patterns which possess strong temporal dependencies-a residual signal with high autocorrelation suggests the unintended retention of valuable pattern information, such as periodic structures.

Consequently, we propose the energy-correlation constraint loss, which effectively suppresses the model’s tendency to memorize anomalies at both the fragment and sequence levels while preserving critical normal components. Residual signals may have high energy due to the possibility of containing more anomalies or noise, aggravating the burden of optimization, so we use the average power of the signal, while the autocorrelation can be measured by its matrix of autocorrelation coefficients $P=\left\{p_{i,j}\right\}\in R^{n\times (l-1)}$ defined in [−1,1].


$$\begin{array}{c} p_{i,j}=\frac{\sum_{t=j+1}^{l}(z_{i,t}-{\overset{―}{z}}_{i})(z_{i,t-j}-{\overset{―}{z}}_{i})}{\sum_{t=1}^{l}(z_{i,t}-{\overset{―}{z}}_{i}{)}^{\text{2}}} \end{array}$$ (4)

Where, $z_{i,j}$ is the observation at time stamp *j* on variable *i* in the residual signal. The energy-correlation constrained loss is then defined as follows:


$$\begin{array}{c} L_{ecc}=\alpha \sum_{k}\frac{\text{1}}{n\times l_{k}}\sum_{j=1}^{l_{k}}({\hat{x}}_{w,i,j}-x_{w,i,j}{)}^{\text{2}}+\beta \frac{\text{1}}{n\times (l-1)}\sum_{i,j}(relu(\left|a_{i,j}\right|-\frac{\phi }{\sqrt{l}}){)}^{\text{2}} \end{array}$$ (5)

Here, $l_{k}$ is the length of the i-th component of the sequence obtained after discrete wavelet decomposition, ${\hat{x}}_{w,i,j}$ is the observation obtained at the j-th timestamp on the i-th reconstructed component after discrete wavelet decomposition. α, β refer to the equilibrium hyperparameter, ϕ is the hyperparameter that controls the maximum tolerance of the autocorrelation coefficient. relu() refers to the *relu* activation function.

### 3.4 Twin frequency attention

Multi-head attention in the time domain often lacks a comprehensive global understanding of the sequence due to the entanglement of diverse components. Consequently, it struggles to explicitly capture the correlations across different frequencies, which motivates the application of attention mechanisms in the frequency domain. In contrast, existing frequency-domain Transformers [23] focus exclusively on global frequency correlations while overlooking the dependencies among more closely linked local information. This limitation can lead to suboptimal reconstruction of certain segments. Furthermore, drastically fluctuating patterns tend to exhibit strong local correlations; however, the aggregation of global relations may attenuate such local dependencies or even introduce irrelevant interference.

To address these challenges, we propose the Twin Frequency Attention mechanism, as shown in Fig 2. Specifically, the local frequency attention divides the series into multiple slices via a sliding window, subsequently applies the Fast Fourier Transform to each slice, and captures locally significant interrelationships through multi-head attention. Conversely, the global frequency attention directly executes multi-head attention across the entire frequency spectrum of the sequence. Finally, the most valuable local or global frequency information is flexibly selected based on the representations from both branches using an adaptive selection strategy. This strategy can dynamically identify the most informative features according to the characteristics of different datasets, and a simple yet effective MLP layer is sufficient to accomplish this task.

**Fig 2:**
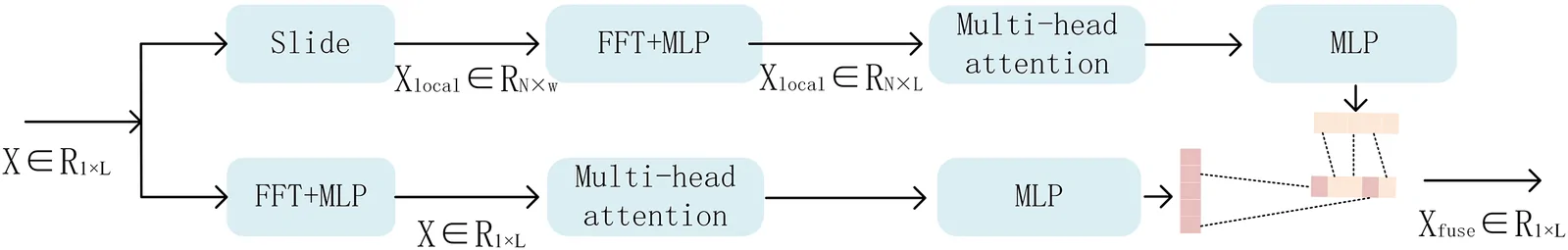
The diagram of twin frequency attention.

### 3.5 Memory storage block

To further enhance the model’s characterization of normal patterns, we introduce a gated update memory storage mechanism as shown in Fig 1. Each memory strip in the memory storage block stores a potential representation of the normal patterns of the sequence, which can adaptively update the memory strips according to the characteristics of the input data to prevent over generalization of anomalies. The memory $M=\left[m_{\text{1}},m_{\text{2}},\cdots ,m_{n}\right]$ is trained to be the potential representation of the normal pattern, $m_{i}$ is the i-th memory strip in *M* and *n* denotes the number of memory strips. Given a query $Z=\left[z_{\text{1}},z_{\text{2}},\cdots ,z_{l}\right]$, the similarity between it and the memory *M* is computed and used as a weight to weight and sum the memory strips:


$$\begin{array}{c} w_{k,i}=\frac{\text{exp}(\frac{\text{1}}{\lambda }(z_{k}*m_{i}))}{\sum_{j=\text{1}}^{n}\text{exp}(\frac{\text{1}}{\lambda }(z_{k}*m_{j}))},\;\hat{Z}=WM \end{array}$$ (6)

Here, $w_{k,i}$ is the similarity between $z_{k}$ and mi, and the weight vector *W* is computed based on the normalized similarity between the query *Z* and the memory *M*. During the memory update phase, the memory storage block is forced to store the potential representation of the most valuable normal pattern through loss function and gating mechanism.


$$\begin{array}{c} v_{k,i}=\frac{\text{exp}(\frac{\text{1}}{\lambda }(z_{i}*m_{k}))}{\sum_{j=\text{1}}^{C}\text{exp}(\frac{\text{1}}{\lambda }(z_{j}*m_{k}))} \end{array}$$ (7)

$$\begin{array}{c} \varphi =Sigmoid(W_{m}M+W_{z}(V*Z)) \end{array}$$ (8)

$$\begin{array}{c} M\leftarrow \varphi (V*Z)+(1-\varphi )M \end{array}$$ (9)

λ is a temperature hyperparameter, φ is the update gate in the memory storage block that flexibly updates the memory strip according to the characteristics of the input data. *W*_*m*_ and *W*_*z*_ are learnable linear mapping matrices. *V* is the similarity matrix consisting of $v_{k,j}$.

The gated update mechanism injects various potential representations of normal data into the memory strips and adaptively determines the degree of update of the strips in a data-driven manner, taking into account the data characteristics of the query *Z* and the memory strips *M*.

### 3.6 Training and abnormal critique criteria

To diminish the difficulty of the time series anomaly detection task, we use sequence reconstruction as a pretext task, and the reconstruction loss is defined as the L_1_ loss between the original sequence and the reconstructed sequence.


$$\begin{array}{c} L_{rec}=\frac{\text{1}}{n}\sum_{i=1}^{n}\|{\hat{X}}_{i}-{X_{i}\|}_{\text{1}} \end{array}$$ (10)

Furthermore, minimizing the entropy [29] of *W* is used as an auxiliary sparsity regularization to constrain the sparsity of the memory weights *W* in Eq. (6) and ensure the retrieval of a finite number of most closely related potential representations in the memory storage block.


$$\begin{array}{c} L_{\text{spa}}=\sum_{k,i}-w_{k,i}log\left(w_{k,i}\right) \end{array}$$ (11)

Combining Eq. (5) energy-correlation constraint loss, Eq. (10) reconstruction loss, and Eq. (11) sparse loss, the final objective loss function is defined as follows:


$$\begin{array}{c} L=\gamma L_{ecc}+\mu L_{rec}+\theta L_{spa} \end{array}$$ (12)

Here, *γ, μ, θ* are hyperparameters that control the weight of each part of the loss function.

For the criteria for judging anomalies, we directly use a two-dimensional deviation-based detection criterion that integrates the input and potential spaces. The L_1_ distance between queries *Z* and *M* in the latent space is defined as the latent space deviation Latent Space Deviation (LSD). Abnormal exhibits a larger deviation in LSD than normal because the potential representation of normal data is stored in the memory storage block. The L_1_ distance between X and $\hat{X}$ in the input space is defined as Input Space Deviation (ISD) and is used as a weight to amplify the difference between normal and abnormal in LSD.


$$\begin{array}{c} L_{LSD}=\frac{\text{1}}{n}\sum_{i=\text{1}}^{n}\|Z_{i}-{M_{i}\|}_{\text{1}},\;L_{ISD}=\frac{\text{1}}{n}\sum_{i=\text{1}}^{n}\|{\hat{X}}_{i}-{X_{i}\|}_{\text{1}} \end{array}$$ (13)

$$\begin{array}{c} score=softmax\left(LSD\left(Z,MastISD(X,\hat{X}\right)\right) \end{array}$$ (14)

For threshold selection, we use the top p% of the combined anomaly scores on the training and validation sets based on the anomaly rate p of the dataset.

## 4. Experiments

### 4.1 Experimental setup

**Implementation details:** We first use the sliding window to slice the sequence into non-overlapping sequences of length 96 as input. 80% of the training dataset is used for training and 20% is used for verification. The initial learning rate is 0.0001, the batch size is 256. The adaptive multi-granularity filtering block is 1, the twin frequency attention block is 1, the plain transformer blocks is 1, the memory storage block is 1, the order of discrete wavelet decomposition is 3, and the memory bar is 10. *α, β, λ* are 0.8, 1.0, and 0.1 separately, and *γ, µ, θ* are 0.6, 0.01, 1. Employing early stopping with the patience of 5 epochs against the validation loss during training. Experiments are implemented through pytorch framework using the NVIDIA RTX 3090 24G GPUs. Widely adopted evaluation metrics, such as Precision, Recall, and F1-score, fail to adequately account for the inherent characteristics of time-series anomalies-specifically, that anomalies often manifest as continuous segments rather than isolated points. This limitation renders these metrics ineffective for evaluating collective anomalies. To address this, we adopt two advanced evaluation protocols: Affiliation-based metrics [50] and Volume Under the Surface (VUS) [51]. The Affiliation metric evaluates performance based on the temporal distance between ground-truth and predicted anomaly events, providing a more nuanced assessment of event-level accuracy. The VUS metric, derived from the Receiver Operating Characteristic (ROC) curve, comprehensively evaluates anomaly detection performance by elevating the assessment from a two-dimensional curve to a three-dimensional surface. Additionally, we employ the Point Adjustment (PA) strategy [10] to ensure fair and consistent comparisons with prior studies. To mitigate the impact of randomness and data variability, all reported experimental results are averaged over three independent runs.

**Datasets:** We evaluated the TFCdetector on seven real-world multivariate time-series datasets and one synthetic dataset. (1) Server Machine Dataset (SMD) is a large dataset that records changes in the state of 28 different machines over a 5-week period at a large Internet company. (2 and 3) SMAP (Soil Moisture Active Passive satellite) and MSL (Mars Science Laboratory) are remotely sensed data generated by two different spacecraft released by NASA, containing 55 instances and 27 instances, respectively. (4) Secure Water Treatment (SWaT) is data collected continuously from 51 sensors and actuators from a scenario of 7 days of normal operation and 4 days of attack. (5) Pooled Server Metrics (PSM) is a dataset containing 26 features collected from within multiple server nodes of eBay. (6) NIPS-TS-Water is a drinking water quality dataset for the ‘Internet of Things’ released in 2018. (7) NIPS-TS-SWAN is a synthesized multivariate time-series dataset extracted from solar photospheric vector magnetograms. (8) Synthetic dataset: we used the publicly available multivariate time series data generation tool (https://github.com/KDD-OpenSource/agots/tree/master) to produce a large synthetic dataset containing four instances with complex dramatic fluctuations, as shown in Fig 3(a). The dataset containing extreme, trend, variance anomalies is used for training and testing, Fig 3(b) shows the instances of the three types of anomalies.

**Fig 3:**
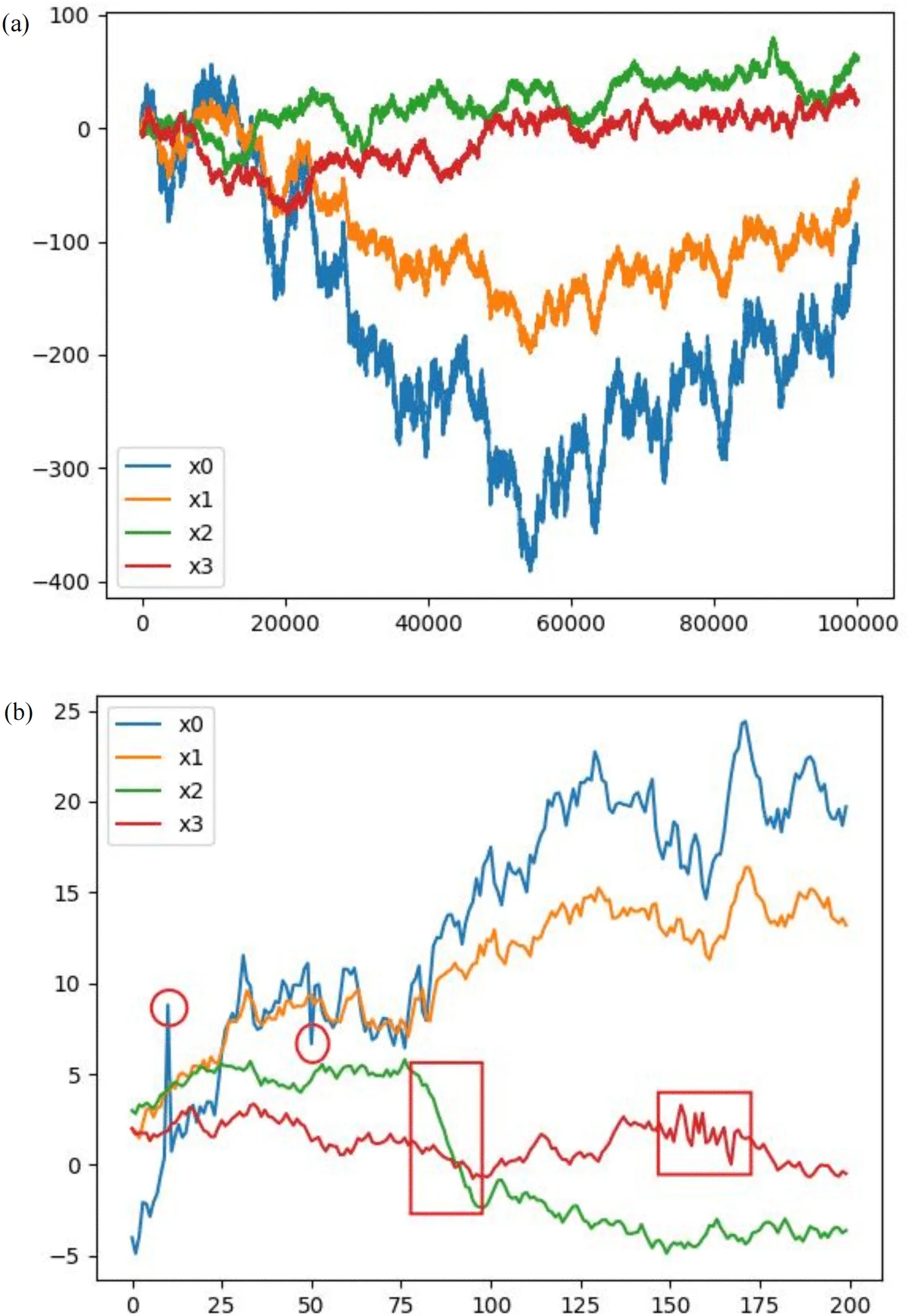
The schematic of the synthetic dataset.

### 4.2 Main results analysis

To comprehensively evaluate the performance of our proposed TFCdetector in the multivariate time series anomaly detection task, we compare the proposed TFCdetector with 12 models, including the classical methods: OCSVM [7], IsolationForest [6], LOF [5], deep learning-based methods: Deep-SVDD [52], ITAD [53], THOC [54], LSTM [12], BeatGAN [14], ImterFusion [13], Anomaly Trans [55], MEMTO [15], TimesMixer++ [56].

Table 1 presents the anomaly detection results of various algorithms across five widely-used multivariate time series datasets. On the SWaT dataset, TFCdetector achieves a substantial improvement in F1-score, outperforming AnomalyTrans by 5.4% and THOC by 10.5%. In contrast, TFCdetector experiences a slight performance degradation on the SMD dataset, which is likely attributable to the inherent limitations of fixed thresholding strategies coupled with the extreme class imbalance present in this dataset. Overall, TFCdetector achieves competitive results across multiple datasets. Notably, even when compared to strong Transformer-based models such as AnomalyTrans and MEMTO, TFCdetector maintains a clear advantage in F1-score. This superior performance can be attributed to the fact that TFCdetector explicitly restricts the model’s learning of anomalies across different frequency sub-sequences, thereby effectively capturing normal patterns and accurately identifying anomalous data.

**Table 1:** Precision (P), Recall (R), and F1 Score (F1) results for 5 real datasets (as %). We reproduce the MEMTO and Anomaly Trans results while adopting the reported performance from Anomaly Trans [55] for the other baselines.

Dataset	SMD			MSL			SMAP			SWaT			PSM		
Metric	P	R	F1	P	R	F1	P	R	F1	P	R	F1	P	R	F1
OCSVM	44.34	76.72	56.19	59.78	86.87	70.82	53.85	59.07	56.34	45.39	49.22	47.23	62.75	80.89	70.67
IsolationForest	42.31	73.29	53.64	53.94	86.54	66.45	52.39	59.07	55.53	49.29	44.95	47.02	76.09	92.45	83.48
LOF	56.34	39.86	46.68	47.72	85.25	61.18	58.93	56.33	57.6	72.15	65.43	68.62	57.89	90.49	70.61
Deep-SVDD	78.54	79.67	79.1	91.92	76.63	83.58	89.93	56.02	69.04	80.42	84.45	82.39	95.41	86.49	90.73
LSTM	78.55	85.28	81.78	85.45	82.50	83.95	89.41	78.13	83.39	86.15	83.27	84.69	76.93	89.64	82.80
ITAD	86.22	73.71	79.48	69.44	84.09	76.07	82.42	66.89	73.85	63.13	52.08	57.08	72.80	64.02	68.13
BeatGAN	72.90	84.09	78.1	89.75	85.42	87.53	92.38	55.85	69.61	64.01	87.46	73.92	90.30	93.84	92.04
ImterFusion	87.02	85.43	86.22	81.28	92.70	86.62	89.77	88.52	89.14	80.59	85.58	83.01	83.61	83.45	83.52
THOC	79.76	90.95	84.99	88.45	90.97	89.69	92.06	89.34	90.68	83.94	86.36	85.13	88.14	90.99	89.54
AnomalyTrans	**88.80**	91.84	90.29	91.62	95.20	93.38	94.09	98.30	96.15	**99.31**	82.67	90.23	97.31	97.63	97.47
MEMTO	88.53	**97.19**	**92.66**	91.60	96.77	94.11	**94.11**	97.98	96.00	94.35	94.22	94.29	97.40	98.20	97.80
TimesMixer++	88.59	84.50	86.50	89.73	82.23	85.82	93.47	60.02	73.10	92.96	94.33	94.64	**98.33**	96.90	97.60
TFCdetector	86.21	90.30	88.21	**92.08**	**99.25**	**95.53**	93.73	**99.27**	**96.42**	94.26	**97.03**	**95.63**	97.36	**98.89**	**98.12**

Recognizing the inherent limitations of point-level metrics in capturing the temporal continuity of anomalies, we further adopt Affiliation-based metrics and Volume Under the Surface to provide a more rigorous, range-aware evaluation. These advanced metrics complement the conventional Precision, Recall, and F1-score by explicitly accounting for the temporal proximity and duration of anomalous segments. Furthermore, since Transformer-based architectures currently represent the dominant paradigm in time series modeling due to their exceptional sequence representation capabilities, we focus our comprehensive multi-metric comparison primarily on these strong baselines. The detailed results are presented in Table 2.

**Table 2:** Multi-metrics results on real-world multivariate datasets. Aff-P and Aff-R are the precision and recall of affiliation metric,respectively. R_A_R and R_A_P are Range-AUC-ROC and Range-AUC-PR, which denote two scores based on label transformation under ROC curve and PR curve, respectively. V_ROC and V_RR are volumes under the surfaces created based on ROC curve and PR curve, respectively. All results are in %, and the best ones are in bold.

Dataset	Method	F1	Aff_P	Aff_R	R_A_R	R_A_P	V_ROC	V_PR
SMD	AnomalyTrans	90.29	70.85	90.19	74.74	71.12	74.98	71.37
	MEMTO	**92.66**	**78.12**	**96.26**	**81.43**	**77.23**	**81.61**	**77.45**
	TFCdetector	88.21	64.72	92.84	76.75	71.89	73.31	68.63
MSL	AnomalyTrans	93.38	83.85	98.97	89.92	87.62	88.83	86.69
	MEMTO	94.11	85.77	98.92	89.45	87.23	88.84	86.70
	TFCdetector	**95.53**	**89.63**	**99.78**	**91.61**	**89.32**	**91.24**	**89.02**
SMAP	AnomalyTrans	96.15	81.05	98.19	95.02	93.16	93.83	92.13
	MEMTO	96.00	79.92	95.31	92.81	91.25	91.86	90.43
	TFCdetector	**96.42**	**84.13**	**99.85**	**96.85**	**94.61**	**96.17**	**94.02**
SWaT	AnomalyTrans	90.23	81.13	63.18	69.61	73.09	69.73	73.19
	MEMTO	94.29	**87.58**	89.95	85.87	85.16	86.06	85.33
	TFCdetector	**95.63**	87.51	**97.62**	**91.21**	**89.82**	**91.65**	**90.19**
PSM	AnomalyTrans	97.47	67.30	84.40	92.69	94.01	89.51	91.72
	MEMTO	97.80	**70.98**	**86.05**	92.91	94.17	**90.11**	**92.17**
	TFCdetector	**98.12**	65.31	83.09	**95.07**	**95.68**	88.18	90.77
NIPS_TS_Swan	AnomalyTrans	73.03	46.59	**4.87**	87.96	94.70	86.06	93.45
	MEMTO	72.77	40.54	4.17	87.94	94.71	86.03	93.45
	TFCdetector	**73.57**	**50.57**	4.63	**87.99**	**94.77**	**86.09**	**93.52**
NIPS_TS_Water	AnomalyTrans	30.07	59.36	88.85	58.03	23.64	57.48	23.02
	MEMTO	32.80	**72.76**	**96.35**	59.40	25.78	59.51	25.94
	TFCdetector	**40.95**	63.46	92.95	**61.89**	**31.14**	**61.44**	**30.78**

The empirical results demonstrate that TFCdetector consistently outperforms Transformer-based models across the majority of metrics on the three standard datasets, exhibiting particularly notable superiority in range-based anomaly detection. To further validate its robustness, we extend the evaluation to the more challenging NIPS-TS-Swan and NIPS-TS-Water datasets, which encompass a significantly broader spectrum of anomaly types. Crucially, by explicitly constraining anomaly representation learning across diverse frequency sub-sequences, TFCdetector effectively refines the characterization of normal temporal dynamics. This architectural design empowers the model to maintain relatively robust and state-of-the-art performance across all metrics, regardless of whether it is evaluated on NIPS-TS-Swan with an extremely high anomaly rate (32.6%) or NIPS-TS-Water with a severely imbalanced, ultra-low anomaly rate (1.1%).

To rigorously evaluate the model’s generalization capability under highly irregular temporal dynamics, we constructed a challenging synthetic dataset characterized by complex, aperiodic variations that lack explicit periodicity-a scenario notoriously difficult for conventional detectors. We benchmark TFCdetector against two state-of-the-art Transformer-based models, Anomaly Transformer [55] and MEMTO [15]. As reported in Table 3, TFCdetector exhibits overwhelming superiority in F1-score on these complex aperiodic series. Remarkably, it achieves substantial relative improvements of 131% and 68.8% over Anomaly Transformer and MEMTO, respectively. This exceptional performance can be directly attributed to our frequency-domain constraint mechanism, which effectively disentangles complex temporal patterns. Furthermore, TFCdetector demonstrates robust capability in identifying subtle contextual anomalies, including trend and variance shifts, even in sequences completely devoid of cyclic patterns.

**Table 3:** Results for multiple indicators on the synthetic dataset, with the best results in bold.

Method	F1	Aff_P	Aff_R	R_A_R	R_A_P	V_ROC	V_PR
AnomalyTrans	9.69	51.35	69.96	61.62	40.41	60.99	38.27
MEMTO	13.25	50.93	66.3	61.69	42.87	61.08	40.60
TFCdetector	**22.36**	**53.98**	**70.63**	**62.03**	**46.22**	**61.57**	**44.79**

### 4.3 Model analysis

To investigate the contribution of key components, we conduct an ablation study, with the results detailed in Table 4. Removing the adaptive multi-granularity filtering module leads to a substantial degradation in metrics such as F1-score, Aff-P, and R-A-R, particularly on the MSL dataset. Furthermore, the twin frequency attention mechanism, which governs the utilization of local and global frequency information, exhibits a more pronounced impact on range-level metrics like R-A-R and V-ROC. This indicates that the selection and fusion of frequency-domain information effectively capture anomalous patterns across varying temporal ranges in the time domain.

**Table 4:** Ablation studies of key modules, the best results are in bold. AMFB refers to adaptive multi-granularity filtering block, TFAB refers to twin frequency attention. The best ones are in bold.

Dataset	AMFB	TFAB	F1	Aff_P	Aff_R	R_A_R	R_A_P	V_ROC	V_PR
MSL	×	×	93.07	83.16	98.37	87.41	85.46	86.59	84.74
	×	√	87.25	75.64	96.22	81.37	79.76	79.55	78.12
	√	×	94.29	85.63	99.15	90.55	88.39	89.53	87.5
	√	√	**95.53**	**89.63**	**99.78**	**91.61**	**89.32**	**91.24**	**89.02**
SMAP	×	×	92.33	80.23	98.78	92.35	90.63	91.24	89.66
	×	√	95.97	79.90	95.53	93.60	91.81	92.67	91.01
	√	×	96.02	81.33	99.45	95.87	93.65	**94.37**	92.34
	√	√	**96.42**	**84.13**	**99.85**	**96.85**	**94.61**	96.17	**94.02**

The proposed twin frequency attention can adaptively select global or local frequency information of valuable sequences, while local windows of different sizes cover different region, affecting the acquisition of sequence information at different granularities. Table 5 explores the impact of different sizes of local windows. As the window length increases, the model performance exhibits a complex float. Especially, the model shows different levels of drastic changes on MSL and PSM, whether it is point-level or region-level metrics. The model achieved relatively optimal results at the window length of 24.

**Table 5:** Effect of local window size in twin frequency attention, best results are in bold.

Dataset	SMAP			PSM			MSL		
Metric	F1	Aff_P	Aff_R	F1	Aff_P	Aff_R	F1	Aff_P	Aff_R
8	96.27	82.82	99.59	98.06	**68.02**	**84.58**	94.77	87.84	99.51
16	96.13	82.04	99.66	97.78	65.53	82.76	95.10	87.63	99.42
24	**96.42**	**84.13**	**99.85**	**98.12**	65.31	83.09	**95.53**	**89.63**	**99.78**
32	96.01	81.43	99.58	97.81	64.22	81.19	93.77	83.97	98.90
40	96.08	81.95	99.47	97.77	64.91	82.97	91.49	81.95	98.11
48	96.30	82.73	99.71	97.72	65.33	83.36	93.93	86.41	99.19

The energy-correlation constraint loss regulates the model’s learning of anomalies at both the fragment and sequence levels. Table 6 illustrates its impact on model performance. The absence of both energy and correlation losses leads to varying degrees of performance degradation across multiple metrics. Specifically, the energy loss enhances the perception of local anomalies, while the correlation loss constrains the model’s fitting to anomalous patterns from a holistic signal perspective. Incorporating either the energy loss or the correlation loss individually yields varying degrees of improvement in metrics such as F1-score, Aff-P, and R-A-R.

**Table 6:** A study of the validity of the energy-correlation constrained loss function, with the best results in bold.

Dataset	Auto_loss	Ene_loss	F1	Aff_P	Aff_R	R_A_R	R_A_P	V_ROC	V_PR
MSL	×	√	93.79	85.03	98.91	90.16	87.98	89.14	87.10
	√	×	92.55	84.17	98.08	89.40	87.15	88.78	86.60
	×	×	87.69	76.26	95.75	80.49	79.34	80.03	78.92
	√	√	**95.53**	**89.63**	**99.78**	**91.61**	**89.32**	**91.24**	**89.02**
SMAP	×	√	96.07	83.29	99.76	95.88	93.70	95.45	93.32
	√	×	96.06	82.30	99.66	95.23	93.08	94.48	92.43
	×	×	96.17	82.79	99.74	95.96	93.78	94.89	92.85
	√	√	**96.42**	**84.13**	**99.85**	**96.85**	**94.61**	**96.17**	**94.02**

TFCdetector has demonstrated exceptional anomaly detection capabilities across most scenarios, but the model inevitably incurs a higher computational burden due to its in-depth exploration of the characteristics of various frequency band subsequences underlying the raw time series. To address this challenge, future work will focus on investigating more lightweight signal decomposition techniques or designing more efficient model architectures through the integration of Mamba.

#### Parameter sensitivity.

We further explored the key parameters in TFCdetector. Fig 4(a) and 4(a1) shows the effect of different transformer blocks, where increasing the depth of the model does not necessarily improve the detection performance well. However, our model achieves the best performance with only 1 layer. The effect of the number of attention heads is investigated on this basis, as shown in Fig 4(b) and 4(b1), which shows that MSL is more sensitive to different numbers of attention heads, and the attention heads is selected to be 8 under a combination of various scenarios. Fig 4(c) and 4(c1) shows the performance under different lengths of input sequences. Since the proposed twin frequency attention includes the attention for localized information, the F1 scores of the model do not decay substantially even as the sequence length increases. Fig 4(d) and 4(d1) shows the effect of the number of memory strips in the memory storage block. The memory strips store potential representations of the normal patterns of the sequences, and the model’s performance is relatively robust as memory strips increases, suggesting that storing a limited number of potential representations of the normal patterns characterizes the intrinsic patterns of the sequences well.

**Fig 4:**
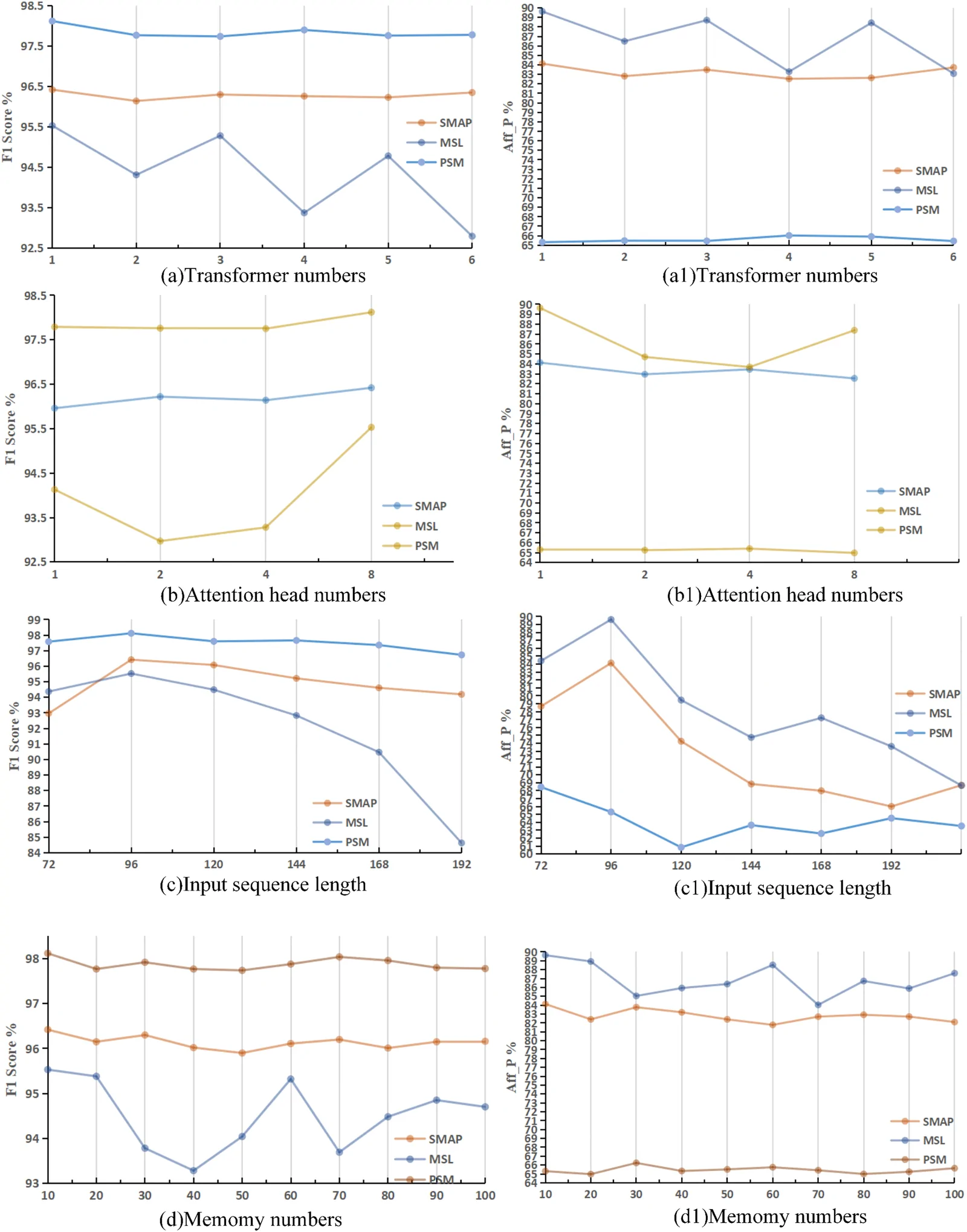
Study on the parameter sensitivity of the main superparameters in TFCdetector.

## 5. Conclusion

For the multivariate time series anomaly detection task, we introduce the TFCdetector from the interrelationships of the anomalie selves, an unsupervised reconstruction-based anomaly detection model. To untangle the entangled series, we project the original time series into mutually non-interleaved sub-band spaces. Within each sub-band, an adaptive multi-granularity filtering module and an energy-correlation constrained loss are employed to suppress the model’s tendency to learn anomalies at both the segment and sequence levels. Furthermore, an adaptively updated memory storage block enhances the expression of normal patterns by leveraging the inherent affinity between normal patterns. Further, the meticulously designed twin frequency attention allows the model to dynamically select task-beneficial local or global information. Despite these advancements, sequence decomposition inherently increases computational overhead and reduces inference speed. Moreover, effectively characterizing sequences with dramatic fluctuations remains a significant challenge. In future work, we plan to explore advanced signal analysis techniques and adaptive representation learning paradigms to more effectively model the complex normal patterns underlying highly volatile and non-stationary sequences.
